# Supplementary material for: End Sequence Analysis Toolkit (ESAT) expands the extractable information from single-cell RNA-seq data
Source: Genome Res. 2016 Oct;26(10):1397–410. doi: 10.1101/gr.207902.116 (PMC5052061; doi:10.1101/gr.207902.116)
Supplement: Supplemental Material [file supp_gr.207902.116_Supp_Materials.tar.gz › ESAT-esat_v0.1/doc/html/dir_c17c136ee8cc04f76d688c588acae175.html]

ESAT: java/jaligner Directory Reference


ESAT


- Main Page
- Related Pages
- Packages
- Classes
- Files

All Classes Namespaces Files Functions Variables Enumerator Pages

- java
- jaligner

jaligner Directory Reference

Directory dependency graph for jaligner:

|  |  |
| --- | --- |
| Directories | |
| directory | formats |
|  | |
| directory | matrix |
|  | |
| directory | util |
|  | |

|  |  |
| --- | --- |
| Files | |
| file | Alignment.java |
|  | |
| file | Cell.java |
|  | |
| file | Directions.java |
|  | |
| file | Markups.java |
|  | |
| file | NeedlemanWunsch.java |
|  | |
| file | NeedlemanWunschGotoh.java |
|  | |
| file | Sequence.java |
|  | |
| file | SmithWatermanGotoh.java |
|  | |
| file | TracebackNode.java |
|  | |


---

Generated on Fri Aug 15 2014 15:11:15 for ESAT by  

 1.8.7
